# Efficacy and safety of imipenem/cilastatin/relebactam (IMI/CS/REL): a meta-analysis of randomized controlled clinical trials

**DOI:** 10.1186/s12879-025-11499-w

**Published:** 2025-09-26

**Authors:** Wenjuan Lei, Yan Duan, Mingyan Xin, Min Tian, Jia Xu

**Affiliations:** 1 Department of Pharmacy, Gansu Provincial Hospital, Lanzhou, 730000 China; 2 Department of Pharmacy, Hunan Provincial People’s Hospital (The First Affiliated Hospital of Hunan Normal University), No.89 Guhan Road, Furong District, Changsha, 410016 China

**Keywords:** Imipenem/cilastatin/relebactam, Efficacy, Safety, Carbapenem resistant gram-negative bacteria

## Abstract

**Background:**

Relebactam is a new inhibitor of class A and class C β-lactamases. The FDA has approved combining IMI/CS/REL to treat some infectious diseases. This study systematically reviews the efficacy and safety of IMI/CS/REL based on existing clinical trials so as to provide a reference for follow-up research.

**Methods:**

Researchers comprehensively searched PubMed, Embase, Cochrane Library, and ClinicalTrials.gov for randomised controlled trials published up to Mar 28, 2025, with “Imipenem/Cilastatin/Relebactam” or “IMI/CS/REL” as keywords, and screened the results according to the proposed exclusion and inclusion criteria. Data extraction was performed on the included randomized controlled trials (RCTs), while the day 28/30 all-cause mortality, clinical response rate, microbiological response rate, the adverse event response rate and drug-related adverse reaction rate were evaluated using R (4.4.3). The evaluation results were expressed in terms of relative risk with 95% confidence limit (RR, 95%CI).

**Results:**

Six RCTs (1606 subjects) were involved in this meta-analysis. The meta-analysis showed no significant differences between IMI/CS/REL and comparators in day 28/30 all-cause mortality (RR = 0.82, 95% CI 0.39–1.72, *P* = 0.10), in clinical response rate during the early follow-up period (EFU) (RR = 1.02, 95%CI 0.96–1.08, *P* = 0.67) and in microbiological response at EFU (RR = 1.02, 95%CI 0.95–1.09, *P* = 0.76). The adverse events (AEs) rate also showed no statistical difference in each study’s arms (RR = 1.02, 95%CI 0.95–1.08, *P* = 0.87).

**Conclusions:**

IMI/CS/REL demonstrates a non-inferior efficacy and safety profile compared to standard comparators in treating carbapenem-susceptible or carbapenem-resistant pathogens. Nevertheless, more robust clinical evidence—especially in resistant, polymicrobial, and pathogen-defined infections—is needed to fully establish its role in real-world practice.

**Supplementary Information:**

The online version contains supplementary material available at 10.1186/s12879-025-11499-w.

## Introduction

Following the discovery of antibiotics, infectious disease treatments have rapidly developed. However, while this is beneficial, people are also facing a new threat: the emergence of drug-resistant bacteria. Indeed, multidrug-resistant (MDR) bacteria such as carbapenem-resistant organisms (CRO) have become prominent [1, 2]. In terms of common bacterial infections, including pneumonia, urinary tract infections, bloodstream infections, tuberculosis, etc., experts across the world have observed a high resistance rate to antibiotics that are commonly used to treat these infections [3–6]. Taking the new coronavirus (COVID-19) infection as an example, the bacterial infection secondary to the new coronavirus infection has greatly increased the mortality rate [7, 8]. Treating drug-resistant bacteria has also increased the medical burden of health organizations globally [9]. In recent years, some new enzyme inhibitors, such as ceftazidime/avibactam, imipenem/cilastatin/relebactam, ceflozane/tazobactam, and meropenem/vaborbactam, have received the FDA’s approval for clinical research or for the treatment of complex intra-abdominal infection (cIAI), hospital acquired pneumonia (HAP), ventilator-associated pneumonia (VAP), and complicated urinary tract infection (cUTI) [10–12]. Relebactam is a non-β-lactam, bicyclic diazabicyclooctane β-lactamase inhibitor of class A (e.g., KPCs) and class C β-lactamases, including carbapenemases. It has been combined with imipenem/cilastatin to deal with infections caused by KPC-producing *K. pneumoniae* and *P. aeruginosa* that express AmpC [13, 14]. Vitro research showed that relebactam can restore susceptibility to imipenem for the majority of imipenem-non-susceptible isolates of *P. aeruginosa* and *K. pneumoniae* tested, as well as some isolates of imipenem-non-susceptible *Enterobacter spp* [15, 16]. Pharmacokinetic studies have demonstrated that IMI/CS/REL excretes primarily through the urinary tract, and that it has a good distribution in lung tissue, which means that physicians can use it to treat pneumonia and urinary tract infections [17, 18]. In addition, IMI/CS/REL has helped to treat cIAI [19]. The IDSA SOC guidelines have demonstrated that patients with febrile neutropenia (FN) may also have Gram-negative infections in cancer patients [20]. IMI/CS/REL exhibited potent activity against Gram-negative infections in patients with cancer [21]. Based on the limited clinical research available, combining IMI/CS/REL (the product was named Recarbrio, Merck & Co) received FDA approval in 2019 for treating cUTI, cIAI, and HAP/VAP caused by susceptible Gram-negative microorganisms in adult patients with limited or no alternative treatment options [22].

At present, clinical research on IMI/CS/REL is ongoing. In view of the therapeutic effect of IMI/CS/REL on drug-resistant bacteria, more clinical indications may be developed in the future. This study systematically reviews the efficacy and safety of IMI/CS/REL based on the existing clinical trials to provide a reference for follow-up research.

## Methods

### Search strategy and selection criteria

This study was conducted according to the guidelines of Preferred Reporting Items for Systematic Reviews and Meta-Analysis (PRISMA) and extension for PRISMA(PRISMA-E 2012) [23, 24]. The study used “Imipenem/Cilastatin/Relebactam” or “IMI/CS/REL” as terms to search PubMed, Embase, and Cochrane Library databases, the deadline was set as Mar 28, 2025.

- The inclusion criteria included: (1) RCTs on the efficacy and safety of IMI/CS/REL, the experimental group was IMI/CS/REL combined with or without any other antibacterial drugs, the dosage of IMI/CS/REL was not limited, and the variety of comparator was not limited; (2) The research subjects were adult patients (Age ≥ 18 years old), and the site of infection was not limited, nor were there restrictions on the types of pathogenic bacteria, regardless of race and gender; research efforts indicate the reported the efficacy outcome including all-cause mortality, clinical response rate and microbiologic response rate at different follow-up periods, the incidence of adverse events (AEs), drug-related adverse events (DRAEs), serious adverse events (SAEs), and serious drug-related adverse events (SDRAEs) were also needed as safety indicators; (3) No language of the research articles was restricted.
- Exclusion criteria included: (1) Non-RCTs; (2) Repeatedly published studies; (3) Study type was non efficacy and safety study; (4) Data were incomplete, lack of specific experimental grouping data, lack of corresponding efficacy and safety outcome indicators, or studies that did not take all-cause mortality, clinical response, microbiologic response, rate of AEs, DRAEs, SAEs, or SDRAEs as clinical endpoint indicators. Two reviewers (Yan and Mingyan) independently completed the literature search and screening. When the reviewers encountered doubts about the screening, a third person would participate in the evaluation and finally discuss whether to include the research.

### Data extraction and quality assessment

The current project designed a research data extraction table. Two reviewers (Min and Jia) extracted the data from the involved RCTs, including the authors’ information, publication year, the non-inferiority margin, clinical trial registration numbers, infection types, IMI/CS/REL dosages, comparators, all-cause mortality, clinical and microbiological responses in different follow-up periods, the incidence of AEs, DRAEs, SAEs and SDRAEs and other specific AEs data. If the data in the paper was incomplete, the researchers would consult the attachments or the experimental data on the clinical trial registration website for supplementary data. The follow-up visit period can be divided into three stages, namely the discontinuation of intravenous therapy (DCIV), early follow-up (EFU, about 7–14 days post-DCIV), and late follow-up (LFU, about 28–42 days post-randomization). The efficacy and safety outcomes of the modified intend-to-treat (MITT, defined as at least one intravenous therapy) population were included for meta-analysis. If there were different doses of experimental groups compared with the same control group in a study, the efficacy and safety data from the different experimental groups were considered. Cochrane Collaboration’s risk of bias tool helped to access the potential sources of bias in RCTs from 7 aspects as followed: (1) Random sequence generation; (2) Allocation concealment; (3) Blinding of participants and personnel; (4) Blinding of outcome assessment; (5) Incomplete outcome data; (6) Selective reporting; (7) Other bias. For each individual domain, the current work classified studies as having low, unclear, or high risk of bias [25].

### Statistical analysis

The current study used the meta and metafor packages of R version 4.4.3 to analyze data. If *I*^2^ > 50%, there was heterogeneity between studies, leading the project to use a random effects (RE) model and Inverse variance method, but otherwise a common effect (CE) model and Mantel-Haenszel method [26]. Relative risk with 95% confidence interval (RR, 95%CI) was used as a summary effect value index. The current study considered the difference statistically significant when *P* < 0.05. Subgroup analysis of efficacy and safety was carried out between different infection types. The results of meta-analysis were represented as a forest plot. This study did not use a funnel plot to assess the publication bias based on the small number of included studies.

## Results

### Study selection and characteristics

**Fig. 1:**
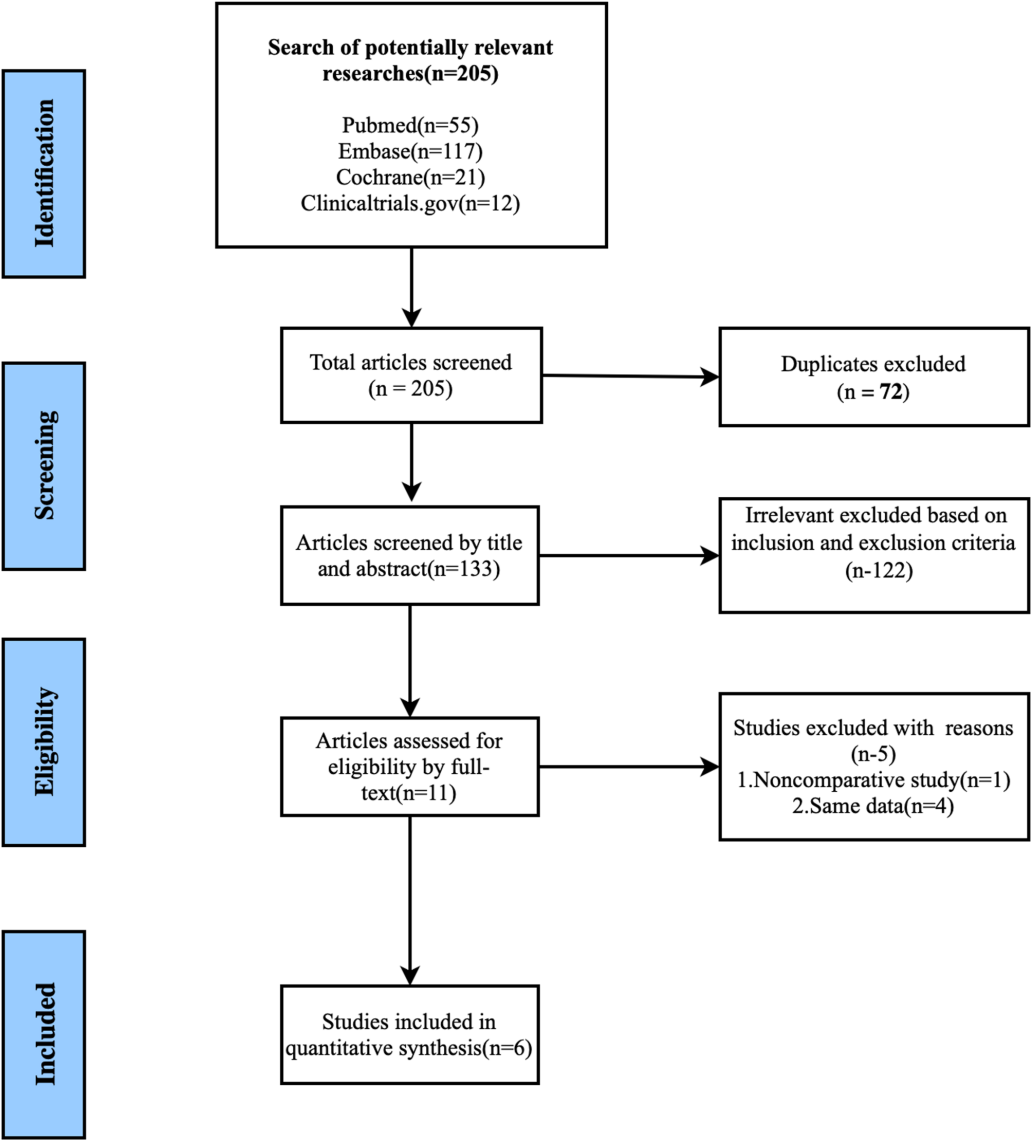
Screening flow chart

The current work identified a total of 205 relevant studies by searching the databases. According to the inclusion criteria and exclusion criteria, the researchers screened the literature. Figure 1 shows the screening flow chart. After excluding 72 duplications, the title and abstract of the studies were reviewed and 122 articles were excluded based on the inclusion and exclusion criteria. The researchers then read the full text of the remaining 11 clinical studies for further screening. They then excluded 4 studies for the same data and a non-controlled clinical study [19]. Roberts 2023 and Titov 2020 had the same clinical trials registration [27, 28]. Three studies emanated from the same RCT study [29–31]. Finally, 6 articles with 1606 patients were included in the analysis [27, 31–35]. Table 1 shows the characteristics of the included 6 RCTs. All the studies were conducted in accordance with principles of Good Clinical Practice and were approved by the appropriate institutional review boards and regulatory agencies. Among the included studies, five were non-inferiority RCTs (prespecified margins: 10–15%), while the remaining one was a descriptive study without prespecified statistical hypotheses [31]. There were four types of infections in this study including, HAP/VAP, cUTIs, cIAIs, and FN. There were two studies with 2 doses regarding an experimental group (IMI/CS/REL 500/500/250 mg and 500/500/125 mg) and comparator group, while the efficacy and safety data were added from the two experimental groups. The main bacterial pathogens included in the study were *Klebsiella pneumoniae*, *Escherichia coli*, and *Pseudomonas aeruginosa.* Only one trial specifically included patients with carbapenem resistant bacterial infection [31].

**Table 1:** Summary characteristics of the included 6 RCTs

Study ID(Author,Year）	Enrolment period	Clinical Trials Registration	Blind method	Non-inferiority Margin	Patients randomized(n)	Infection type	Experiment group			Comparator group			Course of treatment (Day)	Follow up period (Day)	Major outcomes	
							Drug	Dose(mg)	Dosing regimen	Drug	Dose(mg)	Dosage regimen			Effectiveness	Safety
Titov et al.（2020）	2016.01-2019.04	NCT02493764	Double blind	12.5%	537	HABP/VABP	IMI/CS/REL	500/500/250	q6h ivgtt 30min	PIP/TAZ	4000/500	q6h ivgtt 30min	7-14	28	1.Day 28 /30 all-cause mortality (ACM); 2. Favorable clinical response; 3. Favorable microbiologic response.	1.Adverse events；2.Drug-related adverse events; 3. Serious adverse events; 4. Serious drug-related events.
Motsch et al. (2019)	2015.10-2017.11	NCT02452047	Double blind	Descriptive study	47	HAP/VAP/cUTIs/cIAIs	IMI/CS/REL+Placebo (Colistimethate sodium)	500/500/250	q6h ivgtt 30min+q12h ivgtt	IMI/CS+Colistin	500/500+150(300 loading dose)	q6h ivgtt 30min+q12h ivgtt	5-21	28		
Lucasti et al.（2016) [32]	2012.11-2014.08	NCT01506271	Double blind	15%	351	cIAIs	IMI/CS/REL	500/500/250	q6h ivgtt 30min	IMI/CS	500/500	q6h ivgtt 30min	4-14	28-42		
							IMI/CS/REL	500/500/125								
Sims et al.（2017) [35]	2012.12-2015.07	NCT01505634	Double blind	15%	302	cUTIs	IMI/CS/REL	500/500/250	q6h ivgtt 30min	IMI/CS	500/500	q6h ivgtt 30min	4-14	28-42		
							IMI/CS/REL	500/500/125								
Li et al. (2025) [33]	2018.09-2022.07	NCT03583333	Double blind	12.5%	270	HABP/VABP	IMI/CS/REL	500/500/250	q6h ivgtt 30min	PIP/TAZ	4000/500	q6h ivgtt 30min	7-14	28		
Chaftari et al. (2024) [34]	2021.10-2023.8	NCT04983901	open-label	10%	99	in cancer patients with febrile neutropenia	IMI/CS/REL	500/500/250	q6h ivgtt 30min	cefepime	2000	q8h ivgtt 30min	2-14	35-42		
										PIP/TAZ	4000/500	q6h ivgtt 30min				
										meropenem	1000	q8h ivgtt 15min				

*Abbravations*: *HABP* Hospital-acquired bacterial pneumonia, *VABP* Ventilator-associated bacterial pneumonia, *IMI/CS/REL* Imipenem/Cilastatin/Relebactam, *PIP/TAZ* Piperacillin/Tazobactam, *ivgtt* Intravenous drip, *HAP* Hospital-acquired pneumonia, *VAP* Ventilator-associated pneumonia, *cUTIs* Complicated urinary tract infections, *cIAIs* Complicated intraabdominal infections

**Fig. 2:**
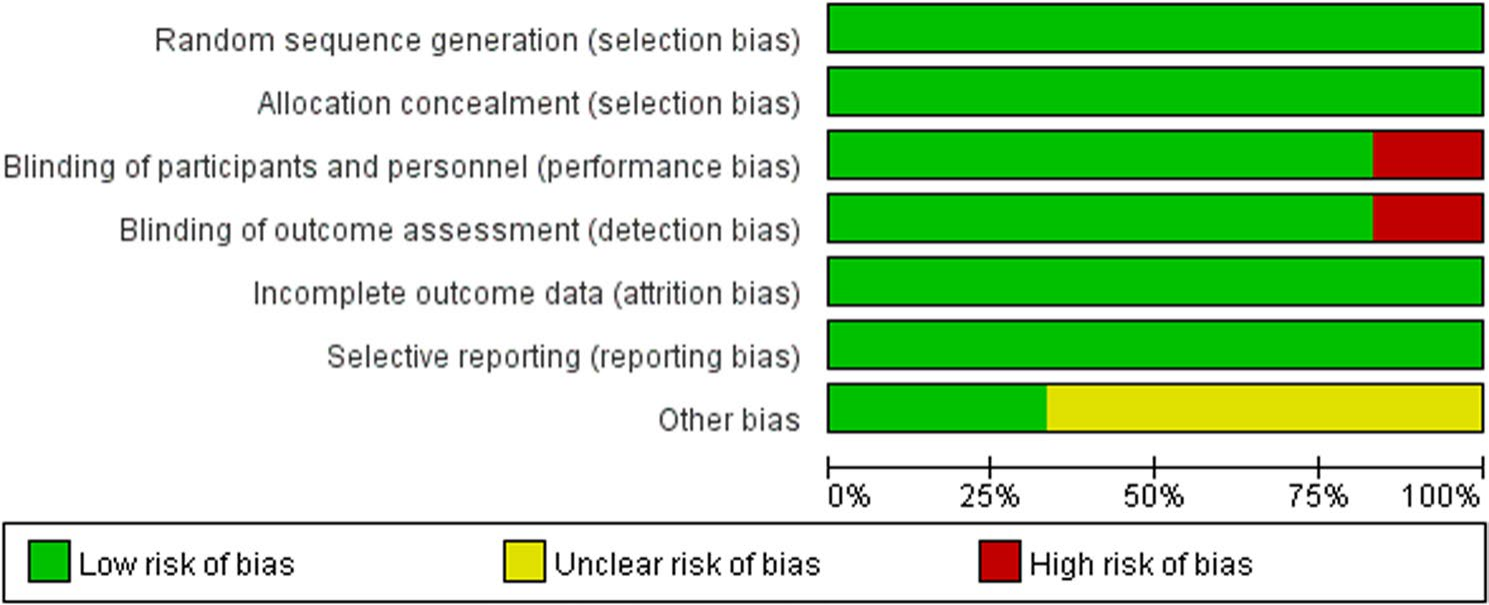
Risk of bias graph

**Fig. 3:**
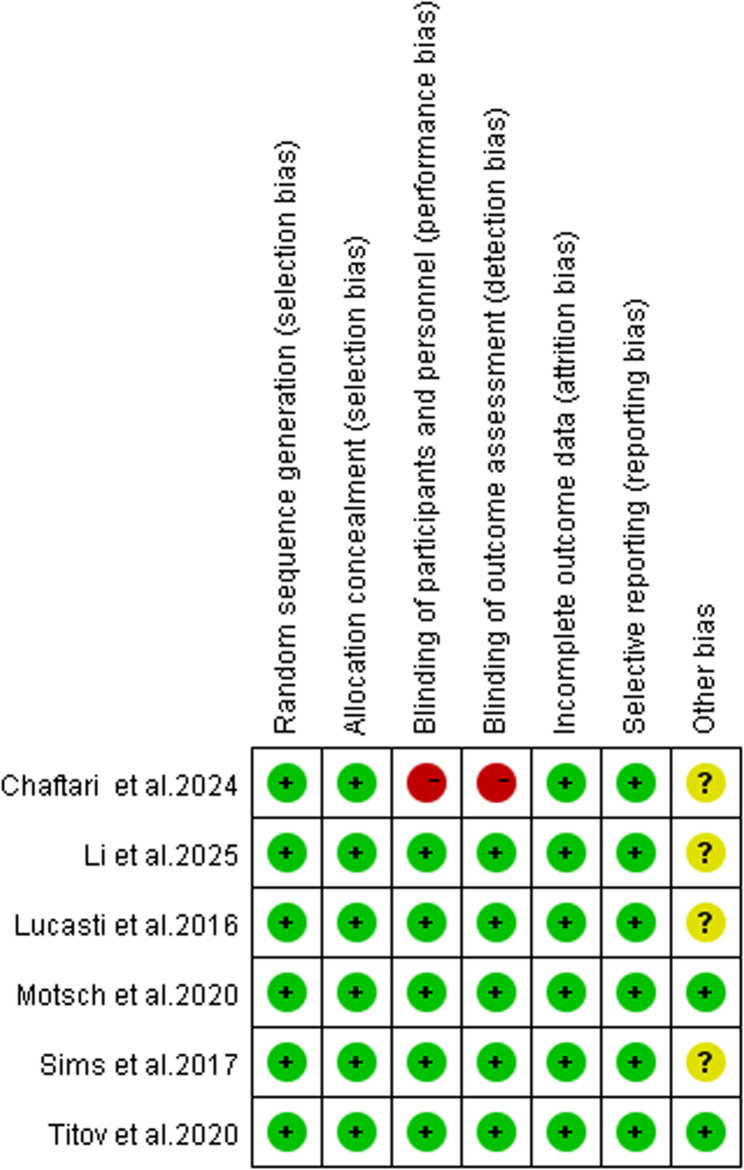
Risk of bias summary

STable 1 and STable 2 exhibit the specific extraction data of efficacy and safety outcomes. The current study used the Cochrane Collaboration’s risk of bias tool to evaluate the risk of bias about the 6 RCTs included in the analysis. The evaluation results are presented in Figs. 2 and 3. All the studies supported by Merck & Co., Inc. Motsch and Titov reported conflicts that the editors consider relevant to the content of the manuscript have been disclosed [27, 31] and other bias was low risk. Chaftari’s study was an open-label clinical study [34].

### The analysis of effectiveness outcomes

Effectiveness outcome data were extracted from the 6 RCTs for meta-analysis, and Table 2 presents the specific results.

**Table 2:** Effectiveness outcomes of IMI/CS/REL with comparators

Outcomes	Follow-up period	No. of involved trials	Total of patients	RR	95%CI	Model of effect	*P* value	Rate of events (*n*/m, %)	
								IMI/CS/REL	Comparator
Day 28/30 all-cause mortality	LFU	4	931	0.82	0.39–1.72	Random	0.60	60/468(12.8%)	71/463(15.3%)
Clinical response	DCIV	5	1087	1.02	0.98–1.07	Common	0.30	477/545(87.5)	464/542(85.6)
	EFU	6	1618	1.02	0.96–1.09	Common	0.46	572/809(70.7)	561/809(69.3)
	LFU	4	817	1.01	0.95–1.08	Common	0.72	336/411(81.8)	330/406(81.3)
Microbiological response	DCIV	4	838	0.99	0.94–1.03	Common	0.55	363/418(86.8)	372/420(88.6)
	EFU	4	1101	1.02	0.95–1.09	Common	0.61	405/544(74.4)	407/557(73.1)
	LFU	3	664	1.01	0.94–1.09	Common	0.76	256/329(77.8)	256/335(76.4)

*Abbreviations*: *IMI/CS/REL* Imipenem/Cilastatin/Relebactam, *DCIV* discontinuation of Intravenous infusion (IV) therapy, *EFU* early follow-up, *LFU* later follow-up, *n/m* Amount of events/number of patients evaluable

Four studies reported all-cause mortality in 931 subjects: three at 28 days and one at 30 days. Overall, there were 60 deaths (60/468, 12.8%) in the IMI/CS/REL group and 71 deaths (71/463, 15.3%) in the comparator group. The RR with 95% CI was 0.82 (0.39–1.72).

Clinical response rate and microbiological response rate were also evaluated in three different stages of the follow-up period (DCIV, EFU, LFU). In the DCIV stage, there were 5 and 4 studies included in clinical response analysis and microbiological response analysis, respectively. A total of 1087 patients participated in the study for clinical response. The clinical response rate of the IMI/CS/REL group was 87.5% (477/545), while that of the comparator group was 85.6% (464/542) and the RR with 95%CI was 1.02 (0.98–1.07). The microbiological response was 86.8% (363/418) in the IMI/CS/REL group, 88.6% (372/420) in the comparator group, and the RR with 95% CI was 0.99 (0.94–1.03).

In the EFU stage, there were 6 and 4 studies included in clinical response analysis and microbiological response analysis, respectively. The clinical response rate in the IMI/CS/REL group was 70.7% (572/809) and in the comparator group it was 69.1% (559/809), while in the RR with 95% CI was 1.02 (0.96–1.09). The microbiological response rate in the IMI/CS/REL group was 74.4% (405/544), the comparator group was 73.1% (407/557), and the RR with 95% CI was 1.02 (0.95–1.09).

In the LFU stage, there were 4 and 3 studies included in clinical response analysis and microbiological response analysis, respectively. The clinical response rate in the IMI/CS/REL group was 81.8% (336/411), in the comparator group it was 81.3% (330/406), and in the RR with 95% CI it was 1.01 (0.95–1.08). The microbiological response rate in the IMI/CS/REL group was 77.8% (256/329), in the comparator group it was 76.4% (256/335), and in the RR with 95% CI was 1.01 (0.94–1.09).

In the meta-analysis of all efficacy outcomes, only the random effects model was used in the analysis of 28/30 all-cause mortality (*I*^2^ = 51.5%), and the others all used a common effect model. There were no statistically significant differences in effectiveness outcomes between the IMI/CS/REL group and the comparator group (*P*>0.05).

**Fig. 4:**
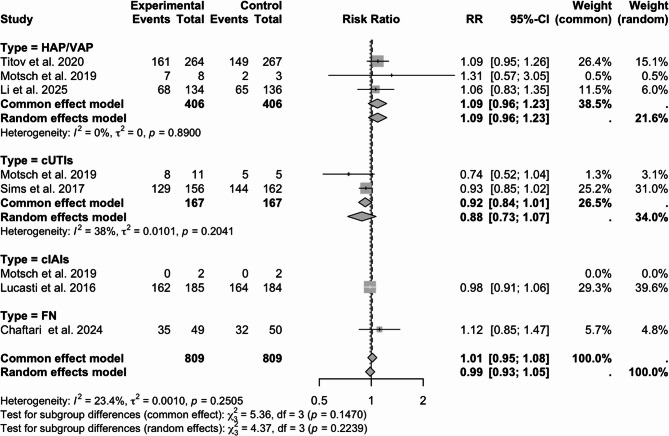
Clinical response at EFU in different infection types

A subgroup analysis was conducted about the clinical response in the EFU stage according to the types of disease; the current study presents the results as a forest plot in Fig. 4. A total of 812 patients with pneumonia (HAP/VAP) participated in the study. The clinical response rate was 58.1% (236/406) in the IMI/CS/REL group and 53.2% (216/406) in the comparator group, the RR with 95% CI was1.09 (0.96–1.23). There were 334 cUTIs patients who participated in the study. The clinical response rate in the IMI/CS/REL group was 82.0% (137/167), while in the comparator group it was 89.2% (149/167), and in the RR with 95% CI was 0.92 (0.84–1.01). 369 patients with cIAIs involved in the analysis, and the clinical response rate in the IMI/CS/REL group was 87.6% (162/185), while the comparator group was 89.1% (164/184), and in the RR with 95% CI was 0.98 (0.91–1.06). 99 patients with FN were involved in the analysis, and the clinical response rate in the IMI/CS/REL group was 71.4% (35/49), while the comparator group was 64.0% (32/50), and in the RR with 95% CI was 1.12 (0.85–1.47).

**Fig. 5:**
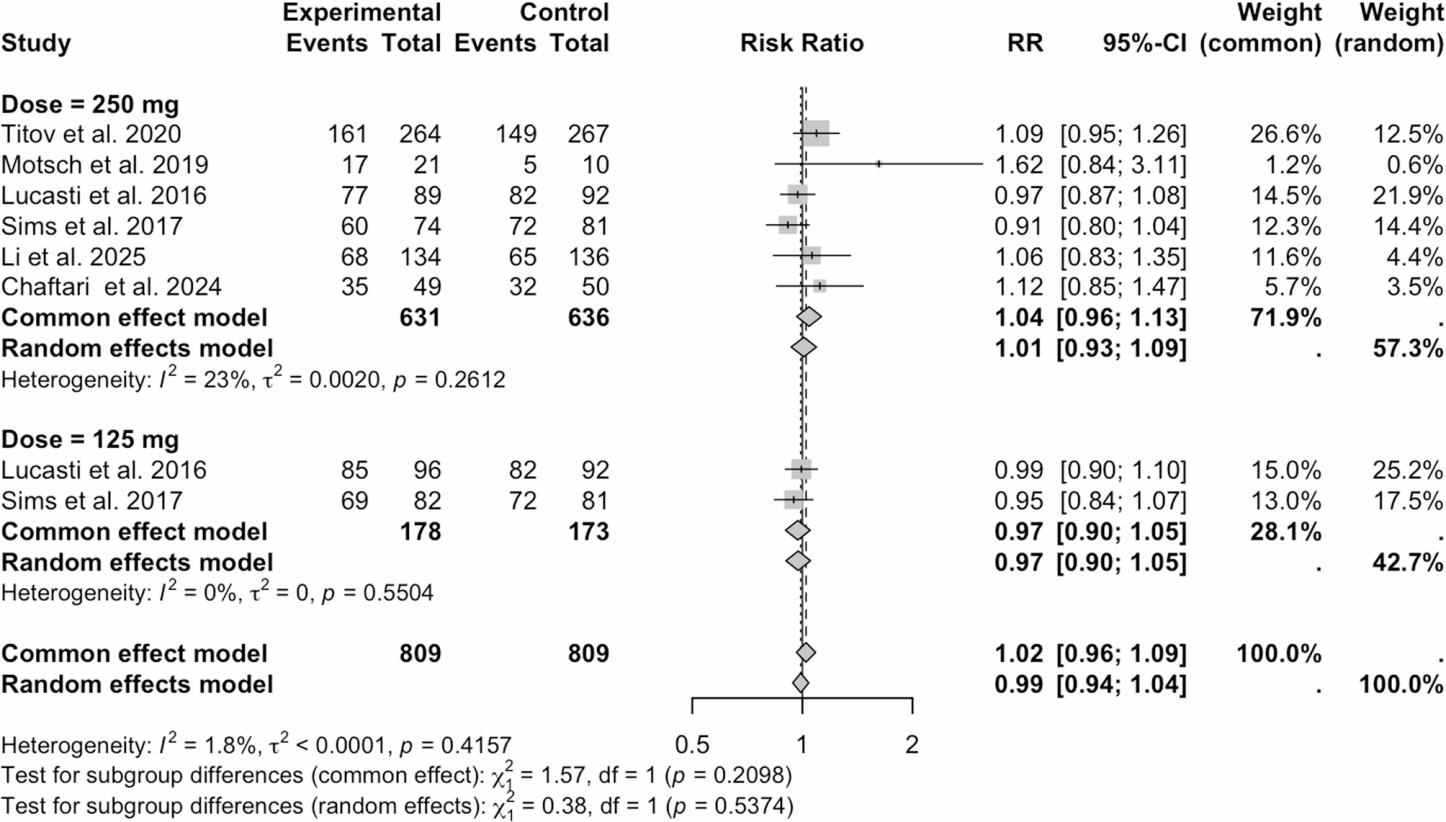
Clinical response at EFU in different doses of IMI/CS/REL

A subgroup analysis was conducted about the clinical response in the EFU stage according to the dose; the current study presents the results as a forest plot in Fig. 5. A total of 631 patients with a dose of 250 mg participated in the study. The clinical response rate was 66.2% (418/631) in the IMI/CS/REL group and 63.7% (405/636) in the comparator group; the RR with 95% CI was 1.04 (0.96–1.13). There were 178 patients with the dose of 125 mg participated in the study. The clinical response rate in the IMI/CS/REL group was 86.5% (154/178), while in the comparator group it was 89.0% (154/173), and in the RR with 95% CI was 0.97 (0.90–1.05).

### Analysis of safety outcomes

Each RCT study conducted safety assessments from four aspects: AEs, DRAEs, SAEs, and SDRAEs. Table 3 shows the specific evaluation results.

**Table 3:** Safety outcomes of IMI/CS/REL with comparators

Outcomes	No. of involved trials	Total of patients	RR	95%CI	Model of effect	*P* value	Rate of events (*n*/m, %)	
							IMI/CS/REL	Comparator
AEs	6	1810	1.02	0.95–1.08	common	0.61	564/911(61.9)	547/899(60.8)
DRAEs	6	1810	1.21	0.94–1.56	common	0.13	118/911(13.0)	95/899(10.6)
SAEs	6	1810	0.93	0.77–1.11	common	0.42	152/911(16.7)	163/899(18.1)
SDRAEs	6	1810	0.71	0.27–1.84	common	0.48	7/911(0.8)	10/899(1.1)

*Abbreviations*: *IMI/CS/REL* Imipenem/Cilastatin/Relebactam, *RR* Relative risk*,* *n/m* Amount of events/number of patients evaluable

In terms of AEs, the incidence of the IMI/CS/REL group was 61.9% (564/911) and that of the comparator group was 60.8% (547/899), while the RR with 95% CI was 1.02 (0.95–1.08).In DRAEs, the incidence of the IMI/CS/REL group was 13.0% (118/911), that of the comparator group was 10.6% (95/899), and in the RR with 95% CI was 1.21 (0.94–1.56). In terms of SAEs, the incidence of IMI/CS/REL group was 16.7% (152/911), and that of the comparator group was 18.1% (163/899), and in the RR with 95% CI was 0.93 (0.77–1.11). In terms of SDRAEs, the incidence of IMI/CS/REL group was 0.8% (7/911) and that of the comparator group was 1.1% (10/899), while in the RR with 95% CI was 0.71 (0.27–1.84). The main reported adverse reactions in the studies were common adverse reactions. Specific adverse reactions were nausea and vomiting in gastrointestinal disturbances. Elevated aspartate aminotransferase, dyspnea, hypoxia, anemia, and hypokalemia had also been reported.

The meta-analysis of all safety outcomes used a common effect model and statistical analysis using the M-H method. The results showed that the safety outcomes were not statistically different between the IMI/CS/REL group and the comparator group (*P*>0.05).

## Discussion

A total of 6 RCTs were involved in this meta-analysis study, which included four infection types and 1606 subjects who participated in the evaluation. In addition to FDA-approved VABPs, cUTIs, and cIAIs, we included a study of cancer patients with febrile neutropenia. Cancer patients with febrile neutropenia were more susceptible to drug-resistant bacteria because they were frequently hospitalized and treated with multiple antibiotics. Therefore, it was important to find new treatment options for such patients. Although we did not conduct a comprehensive analysis of the subject characteristics of each study, the reports of each study explained the absence of differences in the baseline characteristics of subjects between the two study arms.

IMI/CS/REL demonstrated potent in vitro activity against *K. pneumoniae*, *E. coli*, *P. aeruginosa*, multidrug resistant, and restored susceptibility in 70.3% of imipenem-non-susceptible isolates [36, 37]. However, despite this promising microbiological activity, our meta-analysis found no significant differences in clinical and microbiological responses compared to standard comparators. Although there was a tendency to reduce the day 28/30 all-cause mortality, the difference was statistically insignificant. The results of the subgroup analysis also showed that IMI/CS/REL has the same effect as the comparator group in different infection types and doses. The lack of superior efficacy for IMI/CS/REL may be attributed to several factors. First, of the six studies included, only one trial specifically included patients with carbapenem resistant bacterial infection, and the number of patients was limited [31]. Another study described that some patients were infected by carbapenem resistant bacteria, but the proportion of these subjects was quite small [35]. Although IMI/CS/REL showed strong in vitro activity against carbapenem resistant gram-negative bacteria(CR-GNB), including colistin-insensitive strains [15, 38, 39], the low proportion of such patients in clinical trials limits our ability to assess its true clinical efficacy in this setting. Second, as a new type of β - lactamase inhibitor, relebactam can inhibit class A and class C enzymes in β - lactamases, but it may have no or only weak inhibitory effect on other types of enzymes, such as class B (e.g., NDM, VIM and IMP) and class D enzymes (e.g., OXA-48) [13, 40]. Among the included trials, only one conducted enzyme detection on isolated strains of pathogenic bacteria [31], showing a trend toward improved outcomes with IMI/CS/REL. However, this data was limited and had little weight in the overall analysis. Without consistent molecular profiling, its efficacy against specific resistance mechanisms cannot be fully evaluated. Third, the comparators used in the included studies—colistin, IMI/CS, meropenem, cefepime, and piperacillin/tazobactam—are commonly used to treat various infections. Although colistin is commonly used against CR-GNB, its clinical use is constrained by nephrotoxicity [41]. Regarding pathogens in the subjects, very few were CR-GNB. Additionally, the clinical management of infections is influenced by multiple factors, such as infection site, pharmacokinetic/pharmacodynamic (PK/PD) profiles, antimicrobial susceptibility, and the liver and kidney function of the patients. Imipenem retains activity against *Enterococcus spp*., which may provide a therapeutic advantage in treating mixed infections [42, 43]. By virtue of this backbone, IMI/CS/REL may provide broader microbiological coverage than β-lactams lacking anti-enterococcal activity. With rising carbapenem resistance posing critical therapeutic challenges due to limited effective options (e.g., colistin/tigecycline/aminoglycosides), IMI/CS/REL emerges as a promising alternative. Finally, five of the six studies were designed as non-inferiority trials, aiming to demonstrate that IMI/CS/REL is not worse than standard care. None included highly active comparators for resistant infections (e.g., ceftazidime/avibactam). These trial designs may be adequate for regulatory approval, but are limited in establishing superiority in resistant infections.

In terms of safety, the evaluation results showed no significant difference in the incidence of AEs and DRAEs between the IMI/CS/REL arm and the comparator arm. Previous clinical trials among Caucasian populations showed that IMI/CS/REL was mainly excreted through the kidney with good safety. The main adverse reactions were gastrointestinal and discomfort at the injection site [17]. The latest pharmacokinetic and safety study in Chinese healthy subjects also showed that IMI/CS/REL was tolerated well. Similarly, the main adverse reactions were gastrointestinal, while the incidence of nausea was relatively high. Serious adverse reactions included neutropenia and leucopenia caused by multi-dose administration, as well as rash and pruritus [44]. The results of this meta-analysis likewise showed no difference in the incidence of adverse reactions between IMI/CS/REL and the comparator group, and the types of adverse reactions were consistent with the two clinical studies of healthy people. Brown et al. also reported the effect of IMI/CS/REL on renal function from the perspective of the safety of renal function [30]. The results showed that, compared with colistin, IMI/CS/REL had relatively low damage to renal function. Imipenem, a core component, is known to cross the blood-brain barrier and has been associated with seizures, possibly due to GABA receptor antagonism [45]. Interestingly, none of the included studies reported seizure events associated with IMI/CS/REL, though Titov did report one case of generalized tonic-clonic seizure linked to piperacillin-tazobactam [27]. Additionally, Li observed three nervous system-related adverse events—brainstem hemorrhage, cerebral edema, and cerebral hemorrhage—in patients receiving IMI/CS/REL [33]. These findings highlight the importance of close neurological monitoring during IMI/CS/REL therapy, particularly in high-risk populations such as those with renal impairment or a history of epilepsy. Dosage adjustments and individualized risk assessment are crucial to minimizing CNS toxicity. Overall, from the perspective of safety, IMI/CS/REL does not seem riskier to safety than any other comparators.

Several limitations should be taken into consideration when interpreting the results of this meta-analysis. First of all, only six RCTs were included, and only four studies were included in the analysis of some efficacy and safety. The limited experimental data made it impossible to use funnel plot for publication bias analysis. Moreover, due to the lack of consistent microbiological reporting and incomplete β-lactamase characterization across the included studies, we were unable to evaluate the efficacy of IMI/CS/REL against CR-GNB. Furthermore, although there was no heterogeneity in the baseline characteristics of each study among the subjects, and we adopted the principle of randomization, we have observed an unbalanced distribution between the two study arms of subjects who have confirmed a certain infectious pathogen, which may have a certain impact on the efficacy evaluation. These limitations highlight the urgent need for future RCTs that specifically target CR-GNB infections and polymicrobial infections (e.g., cIAIs, particularly those involving *Enterococcus spp.*), incorporate molecular characterization of resistance mechanisms (e.g., KPC, MBL, OXA), and adopt standardized microbiological reporting. Such improvements are essential to more accurately assess the clinical value of IMI/CS/REL in resistant settings.

## Conclusions

In conclusion, the results of this meta-analysis, based on the existing clinical studies, show that IMI/CS/REL was no less effective than any of the comparators in treating pneumonia, complicated urinary tract infection, complicated abdominal infection and febrile neutropenia. There was a trend to reduce mortality, and no apparent higher safety risk. Nevertheless, more clinical trials are needed to confirm safety.

## Supplementary Information

Supplementary Material 1: STable 1. Specific details of the treatment arms and effectiveness outcomes for the included 6 clinical trials.

Supplementary Material 2: STable 2. Specific details of the treatment arms and safety outcomes for the included 6 clinical trials.

## Data Availability

Data is provided within the manuscript or supplementary information files.
