# Supplementary material for: Efficacy and safety of imipenem/cilastatin/relebactam (IMI/CS/REL): a meta-analysis of randomized controlled clinical trials
Source: BMC Infect Dis. 2025 Sep 26;25:1149. doi: 10.1186/s12879-025-11499-w (PMC12465972; doi:10.1186/s12879-025-11499-w)
Supplement: Supplementary file 1 — Supplementary Material 1: STable 1. Specific details of the treatment arms and effectiveness outcomes for the included 6 clinical trials. [file 12879_2025_11499_MOESM1_ESM.docx]

STable 1. Specific details of the treatment arms and effectiveness outcomes for the included 6 clinical trials.

| **Study ID**  **(Author, Year)** | **IMI/CS/REL Dosage(mg)** | **Follow up period** | **Effectiveness outcomes (n/m, %)** | | | | | |
| --- | --- | --- | --- | --- | --- | --- | --- | --- |
|  |  |  | **Day 28/30 all-cause mortality** | | **Clinical response** | | **Microbiologic response** | |
|  |  |  | **IMI/CS/REL** | **Comparator** | **IMI/CS/REL** | **Comparator** | **IMI/CS/REL** | **Comparator** |
| Titov et al.（2020） | 500/500/250 | EFU | 42/264(15.9%) | 57/267(21.3%) | 161/264(61.0%) | 149/267(55.8%) | 146/215(67.9%) | 135/218(61.9%) |
| Motsch et al. (2019) | 500/500/250 | DCIV | 2/21(9.5%) | 3/10(30.0%) | 19/21（90.5%） | 6/10（60%） | — | — |
|  |  | EFU |  |  | 17/21(81.0%) | 5/10(50%) | — | — |
|  |  | LFU |  |  | 15/21（71.4%） | 4/10（40%） | — | — |
| Lucasti et al.（2016） | 500/500/250 | DCIV | — | — | 80/89(89.9%) | 83/92(90.2%) | 81/83(97.6%) | 82/84(97.6%) |
|  |  | EFU |  |  | 77/89(86.5%) | 82/92(89.1%) | 76/78(97.4%) | 78/80(97.5%) |
|  |  | LFU |  |  | 78/89(87.6%) | 79/92(85.9%) | 75/78(96.2%) | 75/78(96.2%) |
|  | 500/500/125 | DCIV | — | — | 88/96(91.7%) | 83/92(90.2%) | 86/86(100.0%) | 82/84(97.6%) |
|  |  | EFU |  |  | 85/96(88.5%) | 82/92(89.1%) | 80/82(97.6%) | 78/80(97.5%) |
|  |  | LFU |  |  | 86/96(89.6%) | 79/92(85.9%) | 80/82(97.6%) | 75/78(96.2%) |
| Sims et al.（2017） | 500/500/250 | DCIV | — | — | 70/74(94.6%) | 81/81(100.0%) | 65/74(87.8%) | 75/81(92.6%) |
|  |  | EFU |  |  | 60 /74(81.1%) | 72/81(88.9%) | 41/74(55.4%) | 51/81(63.0%) |
|  |  | LFU |  |  | 59/74 (79.7%) | 69/81(85.2%) | 44/74(59.5%) | 46/81(56.8%) |
|  | 500/500/125 | DCIV | — | — | 80/82(97.6%) | 81/81(100.0%) | 72/82(87.8%) | 75/81(92.6%) |
|  |  | EFU |  |  | 69/82(84.1%) | 72/81(88.9%) | 51/82(62.2%) | 51/81(63.0%) |
|  |  | LFU |  |  | 65/82 (79.3%) | 69/81(85.2%) | 46/82(56.1%) | 46/81(56.8%) |
| Li et al. (2025) | 500/500/250 | DCIV | 15/134(11.2%) | 8/136(5.9%) | 96/134(71.6%) | 93/136(68.4%) | 46/80(57.5%) | 44/73(60.3%) |
|  |  | EFU |  |  | 68/134(50.7%) | 65/136(47.8%) |  |  |
| Chaftari et al.（2024） | 500/500/250 | DCIV | 1/49（2%） | 3/50（6%） | 44/49（90%） | 37/50（74%） | 13/13（100%） | 14/17（82%） |
|  |  | EFU |  |  | 35/49（71%） | 32/50（64%） | 11/13（85%） | 14/17（82%） |
|  |  | LFU |  |  | 33/49（67%） | 30/50（60%） | 11/13（85%） | 14/17（82%） |

Abbreviations: IMI/CS/REL, Imipenem/Cilastatin/Relebactam; DCIV, discontinuation of Intravenous infusion (IV) therapy; EFU, early follow-up; LFU, later follow-up; n/m, Amount of events/number of patients evaluable.
