# Supplementary material for: Efficacy and safety of imipenem/cilastatin/relebactam (IMI/CS/REL): a meta-analysis of randomized controlled clinical trials
Source: BMC Infect Dis. 2025 Sep 26;25:1149. doi: 10.1186/s12879-025-11499-w (PMC12465972; doi:10.1186/s12879-025-11499-w)
Supplement: Supplementary file 2 — Supplementary Material 2: STable 2. Specific details of the treatment arms and safety outcomes for the included 6 clinical trials. [file 12879_2025_11499_MOESM2_ESM.docx]

STable 2. Specific details of the treatment arms and safety outcomes for the included 6 clinical trials.

| **Study ID (Author, year)** | **IMI/CS/REL Dosage(mg)** | **Safety outcomes (n/m, %)** | | | | | | | |
| --- | --- | --- | --- | --- | --- | --- | --- | --- | --- |
|  |  | **AEs** | | **DRAEs** | | **SAEs** | | **DRSAEs** | |
|  |  | **IMI/CS/REL** | **Comparator** | **IMI/CS/REL** | **Comparator** | **IMI/CS/REL** | **Comparator** | **IMI/CS/REL** | **Comparator** |
| Titov et al.（2020） | 500/500/250 | 226/266(85.0%) | 233/269(86.6%) | 31/266(11.7%) | 26/269(9.7%) | 71/266(26.7%) | 86/269(32.0%) | 3/266(1.1%) | 2/269(0.7%) |
| Motsch et al. (2019) | 500/500/250 | 22/31(71.0%) | 13/16(81.3%) | 5/31(16.1%) | 5/16(31.3%) | 3/31(9.7%) | 5/16(31.3%) | 0/31(0.0%) | 0/16(0.0%) |
| Lucasti et al.（2016） | 500/500/250 | 57/117(48.7%) | 47/114(41.2%) | 16/117(13.7%) | 11/114(9.6%) | 4/117(3.4%) | 8/114(7.0%) | 1/117(0.9%) | 1/114(0.9%) |
|  | 500/500/125 | 55/116(47.4%) |  | 16/116(13.8%) |  | 11/116(9.5%) |  | 0/116(0.0%) |  |
| Sims et al.（2017） | 500/500/250 | 28/99(28.3%) | 30/100(30.0%) | 10/99(10.1%) | 9/100(9.0%) | 3/99(3.0%) | 3/100(3.0%) | 1/99(1.0%) | 1/100(1.0%) |
|  | 500/500/125 | 29/99(29.3%) |  | 9/99(9.1%) |  | 1/99(1.0%) |  | 0/99(0.0%) |  |
| Li et al.(2025) | 500/500/250 | 116/134(86.6%) | 115/13(84.6%) | 27/134(20.1%) | 30/136(22.1%) | 29/134(21.6%) | 21/136(15.5%) | 1/134(0.7%) | 1/136(0.7%) |
| Chaftari et al.（2024） | 500/500/250 | 32/49(65%) | 32/50(64%) | 4/49(8%) | 4/50(16%) | 30/49(61%) | 29/50(58%) | 1/49(2%) | 3/50(6%) |

Abbreviations: IMI/CS/REL, Imipenem/Cilastatin/Relebactam; AEs, Adverse events; DRAEs: Drug-related adverse events; SAEs: Serious adverse events; DRSAEs, Drug-related serious adverse events; n/m, Amount of events/number of patients evaluable.
